# HER2 Expression in Circulating Tumour Cells Isolated from Metastatic Breast Cancer Patients Using a Size-Based Microfluidic Device

**DOI:** 10.3390/cancers13174446

**Published:** 2021-09-03

**Authors:** Cláudia Lopes, Paulina Piairo, Alexandre Chícharo, Sara Abalde-Cela, Liliana R. Pires, Patrícia Corredeira, Patrícia Alves, Laura Muinelo-Romay, Luís Costa, Lorena Diéguez

**Affiliations:** 1 International Iberian Nanotechnology Laboratory, Avenida Mestre José Veiga s/n, 4715-330 Braga, Portugal; claudia.lopes@inl.int (C.L.); alexandre.chicharo@inl.int (A.C.); sara.abalde@inl.int (S.A.-C.); 2 RUBYnanomed Lda, Praça Conde de Agrolongo 123, 4700-312 Braga, Portugal; liliana.pires@rubynanomed.com; 3 Instituto de Medicina Molecular João Lobo Antunes, Faculdade de Medicina, Universidade de Lisboa, Av Prof. Egas Moniz, 1649-028 Lisboa, Portugal; pcorredeira@medicina.ulisboa.pt (P.C.); patricia.alves@medicina.ulisboa.pt (P.A.); luiscosta.oncology@gmail.com (L.C.); 4 Liquid Biopsy Analysis Unit, Oncomet, Health Research Institute of Santiago (IDIS), Complejo Hospitalario de Santiago de Compostela, Trav. Choupana s/n, 15706 Santiago de Compostela, Spain; lmuirom@gmail.com; 5 CIBERONC, Centro de Investigación Biomédica en Red Cáncer, Calle de Melchor Fernández Almagro, 3, 28029 Madrid, Spain; 6 Oncology Division, Hospital de Santa Maria, Centro Hospitalar Lisboa Norte, Av Prof. Egas Moniz, 1649-028 Lisboa, Portugal

**Keywords:** breast cancer, HER2, circulating tumour cells, liquid biopsy, microfluidics, overall survival

## Abstract

**Simple Summary:**

Breast cancer is the most prevalent type of cancer worldwide. By late 2020, there were 7.8 million women alive who had been diagnosed with breast cancer during the previous 5 years. HER2 overexpression in breast cancer is associated with poor prognosis. Existing HER2-targeting therapies significantly improved patient outcomes; still, designing these personalized treatments relies on accurate and comprehensive assessment of HER2 alterations. Frequent HER2 status determination during disease monitoring can be performed using circulating tumour cells (CTCs). Using a novel microfluidic device, we isolated and enumerated CTCs from metastatic breast cancer patients and assessed their HER2 expression in a comparative study with the current gold standard technology. CTCs isolated with our microfluidic technology showed to be a valuable biomarker, and their phenotypical analysis hinted utility to discriminate patient populations, although further validation is needed.

**Abstract:**

HER2 is a prognostic and predictive biomarker in breast cancer, normally assessed in tumour biopsy and used to guide treatment choices. Circulating tumour cells (CTCs) escape the primary tumour and enter the bloodstream, exhibiting great metastatic potential and representing a real-time snapshot of the tumour burden. Liquid biopsy offers the unique opportunity for low invasive sampling in cancer patients and holds the potential to provide valuable information for the clinical management of cancer patients. This study assesses the performance of the RUBYchip™, a microfluidic system for CTC capture based on cell size and deformability, and compares it with the only FDA-approved technology for CTC enumeration, CellSearch^®^. After optimising device performance, 30 whole blood samples from metastatic breast cancer patients were processed with both technologies. The expression of HER2 was assessed in isolated CTCs and compared to tissue biopsy. Results show that the RUBYchip^TM^ was able to isolate CTCs with higher efficiency than CellSearch^®^, up to 10 times more, averaging all samples. An accurate evaluation of different CTC subpopulations, including HER2+ CTCs, was provided. Liquid biopsy through the use of the RUBYchip^TM^ in the clinic can overcome the limitations of histological testing and evaluate HER2 status in patients in real-time, helping to tailor treatment during disease evolution.

## 1. Introduction

Breast cancer is the second most common cancer in the world and one of the leading causes of cancer-related mortality in women, with main incidence between 35 and 75 years old [1,2]. In most of cases, the disease appears sporadically; however, in about 5% of the cases the disease is hereditary with a mutation in coding DNA, leading to a high risk of developing cancer during life [1,3,4,5]. Clinical management and technological advancements allow most primary and early-stage breast cancers to be treated, either by surgery alone or surgery and complementary therapy, achieving an overall 5-year survival rate of 90% [6]. Nevertheless, when cancer spreads and metastasis occurs, the 5-year survival rate drops to 26% [7,8,9]. Research efforts to improve survival rates for metastatic breast cancer (MBC) are currently focused on identifying the molecular heterogeneity of breast cancer and in classifying patients into meaningful subgroups. This classification allows to stratify patients according to prognosis and to define the best therapeutic approach for each group [1].

Nowadays breast cancer can be classified into four molecular subtypes: luminal A, luminal B, basal-like and human epithelial growth factor receptor 2 (HER2) overexpression [1,10,11]. Tumours in the luminal groups present hormonal receptors (oestrogen receptor and/or progesterone, ER+ and/or PR+). Luminal A tumours have high expression levels of hormone-activated genes and low levels of proliferation, being classified as low histological grade (1–2) and good outcome, also with no expression of HER2. On the other hand, luminal B tumours have higher histological grade (2–3), higher proliferation rates and overexpression of HER2 and, consequently, worse prognosis [10,11,12,13]. The other subtypes, basal-like and HER2, are related to the lack of hormonal receptors (ER− and PR−) [10] and associated with aggressiveness and complicated clinical behaviour [1,10]. Basal-like tumours, also known as triple-negative breast cancer, lack also expression of HER2 receptors and represent 15–20% of the cancers, usually having unfavourable diagnosis and outcome, with high histological grade (3), high proliferative index and higher risk of metastases [13,14,15,16,17,18,19]. HER2 tumours display an overexpression of the HER2 protein and associated genes, and they show rapid growth, high histological grade (2–3) and poor behaviour in the therapy [20,21]. HER2 is a member of the epidermal growth factor receptor (EGFR) family of homologous transmembrane receptor tyrosine kinases (RTK) [22,23,24,25] present in 20–30% of breast cancers, having an important role in the progression of the disease, associated with an aggressive disease course and poor survival [22,23,26]. In recent years the survival of HER2 patients has improved with the use of targeted anti-HER2 therapy, achieving survival rates of 43% [27] (OS improved from 20.3 months to 48 months in [28]).

Breast cancer and its expression profile can be studied through tissue biopsy, which aids in subtype classification and grading and is used to define individual therapeutic strategies. If disease relapse happens, treatment decisions are normally still made based on the tissue biopsy of the primary tumour, even when relapse occurs many years after the first diagnosis. A new tissue biopsy is only performed if the tumour site is accessible, when the patient does not respond to therapy. Monitoring of disease progression and evaluation of clinical response relies on biochemical analysis and radiological examination [29]. However, cancer is a heterogeneous and dynamic disease in constant clonal evolution [30], meaning that the tumour characteristics may change over time and across different tumour locations [2,30]. Hence, tissue biopsy often fails to represent intra-tumour and inter-tumour heterogeneity, as it relies on a limited sample of tumour tissue.

Liquid biopsy is a minimally invasive and painless method that provides continuous, reliable and real-time information on the tumour progression through the molecular analysis of circulating biomarkers, overcoming the limitations of the clinical procedures based on imaging and tissue biopsy [31,32]. Liquid biopsy can be used as a complementary diagnostic tool for the study of breast cancer, and it is a promising concept in oncology to investigate tumour heterogeneity, dynamics and progression [33,34,35]. Through the analysis of circulating biomarkers, such as CTCs (circulating tumour cells), ctDNA (circulating DNA) and extracellular vesicles (EVs), liquid biopsy has the potential to access genetic and phenotypic information about the primary and secondary tumours, allowing early diagnosis, accurate prognosis and personalized therapeutics [30,36,37,38]. CTCs are constantly shed from the primary and/or secondary tumour into the bloodstream, and they have a short half-life. Since they present the same phenotype and genotype as the tumour from where they originate, their continuous analysis can provide information about the disease in real-time, allowing a more accurate prognosis [39,40,41]. In addition, since the dissemination of tumour cells is the principal mechanism for metastatic formation, the analysis of CTCs has previously shown to be an ideal approach for early detection of metastasis [42,43].

The study of the presence of HER2 in CTCs can help in providing a more accurate prognosis on the disease evolution, and therefore assist in improving the treatment strategies that may be more effective in lowering CTC burden, reducing the possibility of metastasis formation. Studies showed that some patients have HER2-positive CTCs, even when the primary tumour is HER2-negative, leading to new trials in order to investigate if these patients can benefit from anti-HER2 therapy [44,45]. However, it is not well understood if changes in HER2 status are due to imprecise assessment of the primary tumour or to emergence of new clones of HER2-positive cells [46]. Authors in [47] demonstrated that by using trastuzumab as secondary adjuvant treatment, HER2-positive CTCs were eliminated in patients with HER2-negative primary tumour. The TREAT CTC trial, in which 1317 patients with HER2-negative primary tumour and high levels of CTCs even after neoadjuvant chemotherapy received trastuzumab therapy for 18 weeks, concluded that CTC-based screening is possible in early breast cancer. In addition, it was observed that CTC+ patients presented a higher risk of relapse, and that trastuzumab has no effect on CTCs in HER2- BC [48]. The NSABP B-47 trial supports and confirms this finding, in which trastuzumab has shown to be inefficient in 3270 patients [45,49,50,51,52]. More recent studies, using liquid biopsy systems, have proven able to isolate HER2+ CTCs from breast cancer patients with HER2+ or HER2- tissue biopsy [53]. However, studies to date have failed mainly due to the reduced number of CTCs found in patient samples [54]. CTCs are indeed rare, and their technical isolation is challenging; hence, their application in the clinic has been limited [37,55].

Despite all the methods and technologies available, the CellSearch^®^ system is the only platform for enumeration of CTCs approved by the Food and Drug Administration (FDA) to date [56,57,58]. CellSearch^®^ was designed for the immunomagnetic enrichment, fluorescent labelling and detection of CTCs [57]. Despite the demonstrated clinical validity of CellSearch^®^, its enrichment method causes cell loss affecting the sensitivity of the system. Furthermore, it only selects CTCs expressing EpCAM; thus, other CTC phenotypes are missed, such as mesenchymal and stem cell-like tumour cells that have low levels or no EpCAM expression [58,59,60].

Microfluidics is a technology capable to manipulate fluids using micrometre sized channels [61]. Microfluidic-based separation methods are an attractive alternative to traditional cell isolation technologies due to the use of small sample and reagent volumes with a reduced cost, low contamination issues and no sample-processing steps. By taking advantage of these features, cell loss in samples with low cell concentration can be also reduced, resulting in superior sensitivity and enhanced cell recovery [61,62,63,64]. Several microfluidic devices have been developed for CTCs isolation showing high efficiency. Microfluidic enrichment systems can be classified into two major groups. One group is composed of affinity-based methods, based on positive or negative immune selection; however, these methods end up lacking specialization, since CTCs may not express the biomarkers in use. The other group is based on the physical properties of the CTCs, mainly size-based methods [64,65,66]. Since CTCs are physically distinct from blood cells in many characteristics, including, size and deformability, microfluidic chips for CTC isolation can be made using structures of different geometries for size-based cell filtering.

Among the many size-based methods, it is possible to highlight some such as hydrodynamic and cross-flow filtration, inertial focusing microfluidic systems and systems based on shear-induces diffusion [67,68,69,70]. In the well-known method of inertial microfluidics, the hydrodynamic forces of a fluid are used in the channel in order to focus particles or cells in specific equilibrium positions in a section of the channel, in this case, CTCs [69,71]. Since inertial forces are highly size-dependent, larger cells migrate faster into equilibrium positions, getting on focus, differently from smaller cells [68]. In a more recent size-based method, whole blood was introduced directly in the chip and separated by a buffer flow, and this arrangement triggers the effect of shear-induced diffusion (SID). This effect, allows larger cells to migrate faster into the buffer flow, isolating CTCs from whole blood [69,72].

Several technologies for size-based CTC isolation have been reported with various geometries showing high efficiency. The Vortex chip (Vortex Biosciences) demonstrated high purity (57–94%) but low capture efficiency (up to 37%) of spiked MCF7 breast cancer cells when using diluted blood [73,74]. The Parsortix^TM^ platform (ANGLE) showed an isolation efficiency up to 70% in spiking experiments with cells of a large size; however, among other disadvantages, this system yields low CTC purity (3.1%) [75].

Our group has previously developed and validated different microfluidic devices for the rapid isolation of unfixed CTCs with high efficiency and purity in colorectal and bladder cancer [32,76,77]. The CROSS chip captured CTCs based on their size and deformability with an efficiency of 70% [32]. CTCs were detected in all patient samples and were not observed in the blood of healthy individuals [32,76,77]. Improving on the performance of previously developed systems, our technology processed 7.5 mL of whole blood in just 47 min with enhanced efficiency and purity [32,78].

Due to the numerous advantages of this technology, we designed a study to determine the presence of HER2+ CTCs in a cohort of MBC patients. In parallel, and in order to enhance the compatibility of the system with conventional downstream analysis techniques, the chip was redesigned to fit onto a conventional glass slide. This new generation, the RUBYchip™, was optimised for the processing of breast cancer samples and improved the isolation of breast CTCs based on their size and deformability. The performance of the system was compared blind with the gold standard CellSearch^®^. Finally, the relevance of HER2+ CTCs was studied following the clinical evolution of patients, demonstrating the validity of our system and the overall impact of the implementation of liquid biopsy for patient monitoring, delivering accurate, real-time prognosis and enabling personalized treatment.

## 2. Results

### 2.1. RUBYchip™ Performance Assessment Using Human BC Cell Lines

In order to assess the capture efficiency of the RUBYchip^TM^ for the isolation of breast cancer cells with different phenotypes, human peripheral whole blood samples from healthy donors were spiked with 200 Hoechst-stained cultured cells and processed through the microfluidic chip. Spiking experiments were performed using three cell lines with different levels of HER2 expression, namely MCF-7 (no expression of HER2), MDA-MB-435 (low expression of HER2) and SKBR3 (overexpression of HER2). In addition, four different flow rates were tested for sample processing: 100, 120, 140 and 160 μL/min.

**Figure 1:**
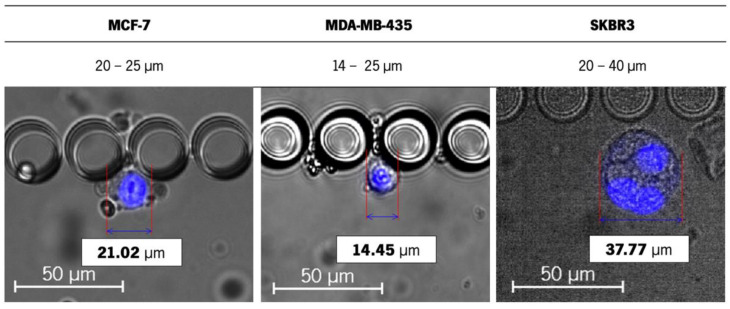
Representative image of cells of three different cell lines trapped inside the RUBYchip™ at the spiking assay. MCF-7, MDA-MB-435 and SKBR3 estimated sizes are 21.02, 14.45 and 37.77 μm, respectively. The images were acquired and observed with a 20× objective. The scale bars correspond to 50 μm.

Since the isolation capacity of the RUBYchip™ relies on the cancer cell size and deformability, the size and morphology of the cell lines were assessed inside the microchip. The average size of the tested cells varied from 14 to 40 μm (MDA-MB-435 being the smallest in size and SKR3 the largest) (Figure 1), and some events of larger dimensions were observed, from 50 to 60 μm, possibly due to different stages of the cell cycle and likely in mitosis. Still, within this range of cell size, there was no significant variation in the capture efficiency across cell lines.

**Figure 2:**
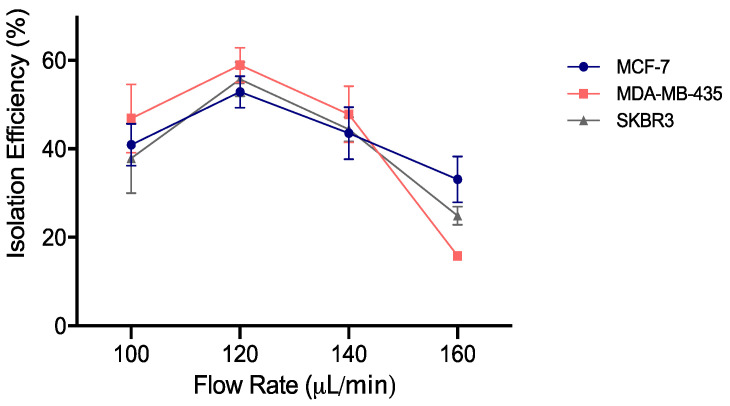
Graphical representation of the isolation efficiency of the RUBYchip™ at four different flow rates, with three different cell lines, MCF-7, MDA-MB-435 and SKBR3. The highest results were achieved at 120 μL/min, for all the cell lines, with isolations of 53%, 59% and 56% for MCF-7, MDA-MB-435 and SKBR3 breast cancer cells, respectively, showing consistency of the results.

The highest capture efficiency was achieved at 120 μL/min, consistently for all the cell lines tested, such that the RUBYchip™ was able to isolate an average of 53%, 59% and 56% of spiked MCF-7, MDA-MB-435 and SKBR3 breast cancer cells, respectively (Figure 2). The capture efficiency decreased in all the others flow rates tested. Considering these results, 120 μL/min was determined as the optimal flow rate to be used for future processing of breast cancer patient samples. This flow rate allowed a fast sample processing, making it possible to process 7.5 mL of whole blood in 63 min.

### 2.2. Cell Staining and Analysis Criteria

In order to optimize the antibody conditions to be used for patient samples in the RUBYchip™, several immunocytochemistry (ICC) experiments were performed in cultured adherent cells as well as cell suspensions processed inside the microfluidic device. Negative controls were included using samples from healthy volunteers.

The ICC experiments were carried out using MCF-7, MDA-MB-435 and SKBR3 cell lines, as well as peripheral blood mononuclear cells (PBMCs). According to the literature, SKBR3 cells exhibit cytoplasmatic cytokeratin (CK) expression and HER2 overexpression in the cell membrane; MCF7 cells express CK but not HER2; MDA-MB-435 do not express any of the selected biomarkers. PBMCs were labelled using CD45, which was used as an exclusion criterion to evaluate CTCs [79,80,81].

**Figure 3:**
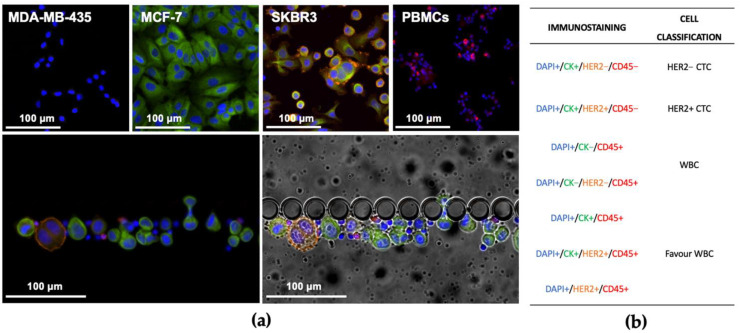
(**a**) Images obtained from the immunocytochemistry experiments in cultured adhered cells (top images) and in cells released into solution inside the device (bottom images). ICC experiments were performed using MCF-7, MDA-MB-435, SKBR3 and PBMCs. Each cell line was tested individually in glass slides (top); additionally, a cell mixture of the different cell lines was spiked in healthy whole blood samples and processed in the device (bottom). MCF-7 cells stained strongly for CK, with cytoplasm localization; SKBR3 cell membrane stained positively for HER2; most PBMCs stained for CD45. (**b**) For further image analysis, CTC classification was established according to the combination of the presence and absence of the selected biomarkers, and CD45 is used as CTC exclusion criteria.

Immunocytochemistry demonstrated that this staining cocktail can discriminate between the three different cell lines and the negative control, as follows: MDA-MB-435 are DAPI+/CK−/HER2−/CD45−, MCF-7 are DAPI+/CK+/HER2−/CD45−, SKBR3 are DAPI+/CK+/HER2+/CD45− and PBMCs are DAPI+/CK−/HER2−/CD45+. These results are in accordance with previous findings [82,83,84,85]. Once immunostaining specificity was verified, the CTC classification for patient samples analysis was established as shown in Figure 3. Once the antibody cocktail and conditions for the immunofluorescence signal acquisition were thoroughly optimised using either culture adherent cells in a well-plate, or a suspension of cultured cells into the device, optimal antibody dilutions were redefined using a patient sample and established to be applied in the analysis of further patient samples, namely 1:200 for CK, 1:106 for HER2 and 1:100 for CD45.

### 2.3. Patient Recruitment and Clinical Characteristics

A total of 15 eligible patients diagnosed with MBC, either de novo or after recurrence, and followed at Hospital de Santa Maria, were recruited to participate in this study. The patients entered the study by the end of 2018, and collections were made from that time.

The main clinicopathological characteristics of the patient cohort are presented in Table 1. Regarding this cohort, the average age of the patients at metastases diagnosis was 47 years; 80% of those patients were less than 60 years old and 20% were 60 or older. The longest time elapsed between the first diagnosis and the metastasis diagnosis was of 13 years (patient 6) and the shorter time was of 0 months (patient 10), with the metastasis diagnosis coinciding with the first diagnosis, averaging 68 months until disease relapse occurred (6 years). Metastases were found in different sites, and the highest incidence was in visceral organs (40%), followed by bone (33.3%) present in five patients. Four patients had metastases in multiple sites simultaneously (26.7%), namely, bone and lung (1 patient, 6.7%), lung and lymph node (1 patient, 6.7%), liver and cutaneous (1 patient, 6.7%), and one patient had cutaneous metastases (6.7%).

The cohort can be divided into five different groups according to their breast cancer subtype. A total of 46.7% of the patients had ER+/PR+/HER2− tumours, which is the most common subtype, followed by ER+/PR−/HER2− occurring in 20% of the patients, ER+/PR+/HER2+ and ER+/PR−/HER2+ occurring in 13.3% of the patients each, and triple-negative breast cancer (ER−/PR−/HER2−) diagnosed in 6.7% of the patients. These different subtypes of BC were included in the study so that it was possible to analyse and cover all BC patients, as well as to analyse the presence of HER2 in the CTCs of patients with different primary tumour subtypes. Most patients were diagnosed with disease relapse (93.3%), but 6.7% or were diagnosed de novo with metastatic disease.

Overall cancer treatment was administrated following the institutional guidelines and in compliance with the international oncology society guidelines.

At the moment of the first collection, most of the patients received systemic therapy, except one patient (patient 12, with TNBC) who was treated with radiotherapy. Five patients (33.3%) received systemic chemotherapy, and eight patients were treated with hormone therapy (53.3%). In addition, three patients (20.0%) with HER2-positive primary tumours were treated with anti-HER2 therapy (alone or combined with chemotherapy), and five patients (33.3%) received cyclin inhibitor therapy combined with hormone therapy. Bone target therapy was also administrated in four patients (26.7%) who had bone metastases.

### 2.4. Isolation of CTCs Using the RUBYchip™ and CellSearch^®^

The performance of the RUBYchip™ using patient samples was next evaluated by comparing this technology with the gold standard and FDA-approved system for CTCs enumeration in the clinic, CellSearch^®^.

For each patient, two tubes of 7.5 mL of peripheral blood were collected and processed in parallel using either technology. Collection for each patient took place at two time points: at baseline, before starting systemic treatment, and at monitoring follow-up, after 12 weeks of ongoing treatment. After cell isolation, a randomized blind sample analysis was performed, and results obtained from both technologies were compared. In order to reduce inter-laboratory variability in CTC enumeration and provide validation against the gold standard methodology, all CTC image galleries produced by the analysis of RUBYchip™ were also independently interrogated by a highly experienced operator, responsible for a liquid biopsy unit at a reference laboratory.

The number of CTCs identified by the RUBYchip™ and the CellSearch^®^ were compared head-to-head including all 30 samples, without discrimination of time-points. In addition, to further investigate the CTC count evolution and its association with disease progression, baseline and follow-up collections were compared for each patient.

**Figure 4:**
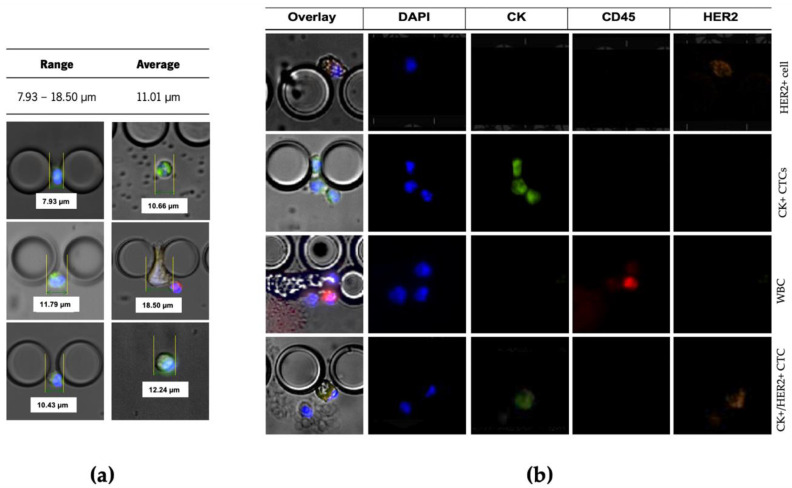
(**a**) Representative image of CTCs trapped inside the RUBYchip^TM^. Size was estimated using measurements of different patient samples. CTCs size range is from 7.93 μm up to 18.50 μm. (**b**) Representative image of the different types of cells trapped inside the chip. Isolated cells trapped between pillars of the chip were stained with Anti-Cytokeratin pan-FITC, Cyanin5 Anti-Human CD45, DyLight 550 Anti-ErbB2/HER2, and the nuclear marker DAPI. First, a DAPI+/CK−/CD45−/HER2+ captured cell is shown. In the second line, three captured CK+ CTCs are represented, DAPI+/CK+/CD45−/HER2−. The third line shows a white blood cells, DAPI+/CK−/CD45+/HER2−. Finally, a DAPI+/CK+/CD45−/HER2+ is shown. The images were acquired and observed with a 20× objective.

The cells isolated between pillars of the microfluidic device were stained with Anti-Cytokeratin pan-FITC, Cyanin5 Anti-Human CD45, DyLight 550 Anti-ErbB2/HER2 and the nuclear dye DAPI. Only DAPI+/CK+/CD45−/HER2+ and DAPI+/CK+/CD45−/HER2− cells were considered CTCs (Figure 4b). These trapped cells’ estimated cell size range was from 7.93 μm to 18.50 μm (Figure 4a).

**Figure 5:**
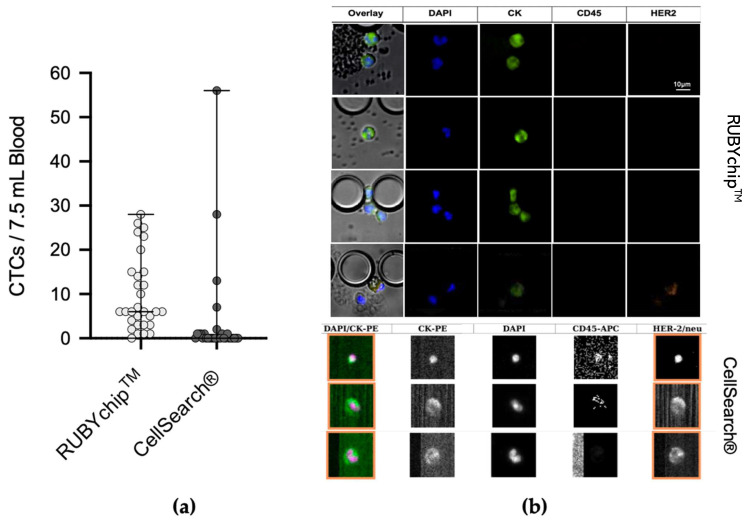
(**a**) Comparative scatterplot demonstrating the enumeration of CTCs using the RUBYchip™, in light grey versus the CellSearch^®^ system, in dark grey, for all the 30 samples analysed. (**b**) Representative images of captured cells using both technologies. CellSearch^®^ on the bottom, and RUBYchip™ on the top. RUBYchip™ technology allows to obtain high-resolution images, and image quality is clearly superior when compared to CellSearch^®^. Isolated cells trapped between pillars of the RUBYchip™ were stained with Anti-Cytokeratin pan-FITC, Cyanine5 Anti-Human CD45, DyLight 550 Anti-ErbB2/HER2 and the nuclear marker DAPI.

The comparative study of the 30 MBC samples demonstrated considerable differences between both technologies in CTC enumeration. The average number of CTCs isolated by the RUBYchip™ at baseline and follow-up samples was 12 and 8, respectively, in considerable contrast to the CellSearch^®^, which isolated an average of 2 CTCs at baseline and none at follow-up samples (Figure 5). Overall, RUBYchip^TM^ was able to isolate CTCs with higher efficiency than CellSearch^®^, up to 10 times more, averaging all 30 samples (*p* < 0.0002, Wilcoxon test). Strikingly, the RUBYchip™ was able to detect CTCs in 97% of all 30 samples examined as opposed to only 37% on CellSearch^®^, which indicated the superior efficiency of the first. In addition, considering the threshold established for poor disease progression at 5 or more CTCs, the CellSearch^®^ counts were below 5 for 26 of the samples (87%), while the RUBYchip™ counted at least 5 CTCs in 21 of the samples (70%), allowing a more accurate evaluation of the disease prognosis for each patient (Table 2).

Representative immunofluorescence micrographs of CTCs from both the CellSearch^®^ and the RUBYchip™ are presented in Figure 5. As shown, image quality obtained with the RUBYchip™ technology considerably improved compared to CellSearch^®^, allowing for easier, faster and more reliable analysis

### 2.5. HER2 Status Assessment Using Liquid Biopsy and Tissue Biopsy

**Figure 6:**
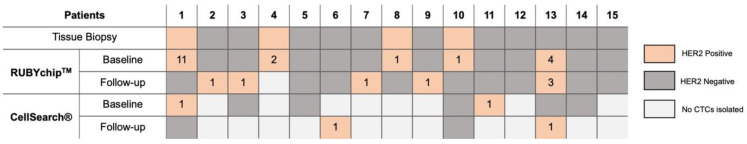
Representative scheme on the HER2 expression status on the primary tumour by tissue biopsy and on the CTCs at two different collection time-points, baseline (before starting systemic therapy) and follow-up (after 12 weeks of systemic treatment), by both technologies, RUBYchip™ and CellSearch^®^. The HER2+ CTC events are represented in orange, HER2− CTC in grey, and in white no CTCs were isolated. The number of HER2+ CTCs is represented at the respective collection moment, for each technology.

HER2 is an established therapeutic target in breast cancer; hence, adequate use of targeted therapy depends on accurate assessment of HER2 status. To further evaluate the value of the HER2 analysis in CTCs isolated using a microfluidic approach, as a tool for a real-time diagnosis and follow-up, the presence of HER2-positive CTCs in the patient samples was assessed by both technologies. Besides HER2+ CTCs (DAPI+/CK+/HER2+/CD45−) enumeration by RUBYchip™ and CellSearch^®^, liquid biopsy findings were compared with the histopathological assessment of HER2 status from the tissue biopsy of the primary tumour (Figure 6). DAPI+/CK−/HER2+/CD45− events were not considered for CTC enumeration.

Overall, also in the detection of HER2-positive CTCs, RUBYchip™ was considerably more efficient, as it was able to isolate HER2+ CTCs in nine patients (60%), while CellSearch^®^ only did in four patients (26.7%). It is noteworthy to mention that results obtained with the RUBYchip™ were in agreement with tissue biopsy assessment of HER2 expression in the majority of cases at the baseline analysis, 14/15 patients (93.3%), which is the closest time-point to the tissue biopsy. In opposition, CellSearch^®^ was largely unable to confirm or refute tissue biopsy HER2 status assessment at baseline, since it yielded inconclusive results (no CTCs) in eight cases (over 50%), additionally differed regarding two patients and agreed in 5 patients only. Moreover, at follow-up collection, rate of inconclusive HER2 testing with CellSearch^®^ increased to 73.3%, as the technology was not able to detect any CTC in 11 of the 15 patients. The low efficiency of CellSearch^®^ or the presence of CTCs with low expression of EpCAM were assumed to be accountable for such results. In total, CellSearch^®^ was unable to detect over 1 HER2+ CTC per sample, while in contrast RUBYchip™ was able to detect on a range of 1 to 11 HER2+ CTCs.

Interestingly, results from both technologies combined demonstrate that liquid biopsy is able to detect more HER2 positivity cases than the tissue-based approach; 11 patients had HER2+ CTCs detected (73.3%) as opposed to 4 patients that tested positive for HER2 in primary tumour tissue assessment (26.7%). This result might be indicative of the sampling limitations in conventional tissue biopsy that fails to reflect tumour heterogeneity, or simply due to disease progression.

Easily repeatable sampling for frequent monitoring is an accepted advantage of liquid biopsy to access therapeutic resistance and base decision-making during treatment cycles. Interestingly, the group of patients (Patients 1, 4, 8 and 10) diagnosed as having HER2+ tumours that were subjected to anti-HER2 therapy, in between the two moments of blood collection, were confirmed positive for HER2+ CTCs at the baseline; however, they showed no HER2+ CTCs at the follow-up, suggesting the success of the anti-HER2 targeted therapy. In contrast, CellSearch^®^ only detected HER2+ CTCs in one of these four patients.

### 2.6. Correlation of Clinicopathological Information with CTC Enumeration and Characterisation

To further evaluate the implications of CTC analysis in MBC setting, it was evaluated whether CTC enumeration and HER2 status findings were associated with relevant clinicopathological characteristics, including BC subtype, HER2 status in primary tumour, administrated treatment/therapeutic options and clinical stage.

It was observed how the number of isolated CTCs evolved during the course of the disease, taking into account the therapy administered in the elapsed time. The number of CTCs isolated by either technology in the 15 metastatic breast cancer patients at the two time points is presented in Table 3. Considering the RUBYchip™ results, five patients (33.3%) had increased CTC counts at follow-up when compared to the baseline collection, namely, patients 1, 6, 11, 12 and 13. HER2+ CTCs were detected by the RUBYchip™ in patients 1 and 13, as referred in the previous section. Patient 1, which was diagnosed as having a HER2+ tumour, received anti-HER2 therapy after baseline collection and, although an increase in the total number of CTCs was observed, the number of HER2-positive CTCs decreased. Patient 13 was diagnosed as having a TNBC tumour and had an increase of 18 CTCs at follow-up (3× higher than baseline). A considerable increase in the total number of CTCs at follow-up was also observed in patients 11 and 12, this last one with TNBC, who did not receive systemic therapy. All the other patients showed a decrease in the number of total CTCs. Most noticeable cases of decreased number of CTCs at follow-up, compared to baseline, were observed in patients 3 (26× less CTCs), 4 (from 24 to 0 CTCs) and 5 (from 17 to 0 CTCs).

### 2.7. Prognostic Value of CTC Enumeration

**Figure 7:**
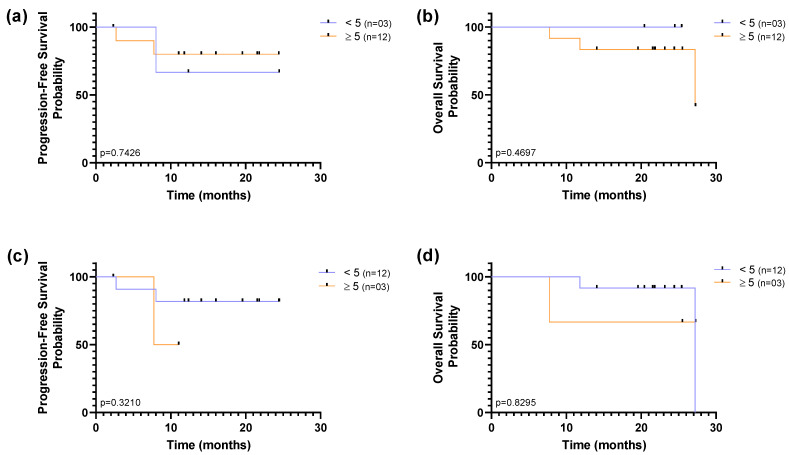
Kaplan–Meier curves of progression-free survival (PFS) and overall survival (OS) according to CTC count (≥5 CTCs/7.5 mL of blood), using (**a**,**b**) RUBYchip™ and (**c**,**d**) CellSearch^®^ for isolation of CTC in MBC patients. PFS and OS were calculated from the time of the baseline blood collection. Differences in survival curves were determined using the log-rank test.

To assess the prognostic value of CTCs counts in this cohort, the 15 enrolled patients were divided into baseline CTC < 5 and CTC ≥ 5 groups, which is the established threshold to prognostic favourable and unfavourable CTC-counts in mBC according to the FDA approved procedure. Kaplan–Meier survival curves to determine the PFS and OS for the two groups using both CTC isolation technologies are shown in Figure 7. Out of the 15 patients, 80% had ≥5 CTCs detected using the RUBYchip™, whereas only 20% had been detected using CellSearch^®^.

**Figure 8:**
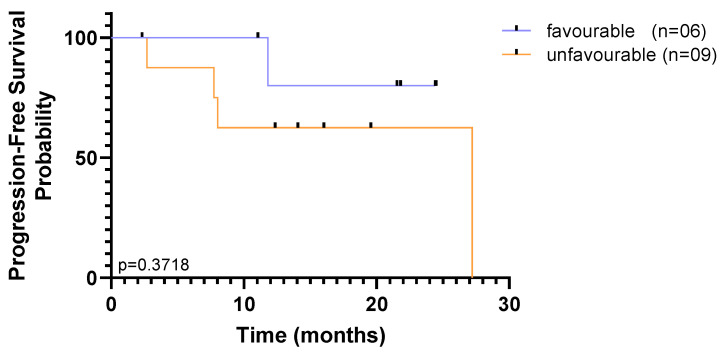
Kaplan–Meier curves of progression-free survival (PFS) according to favourable or unfavourable CTC count (cut-off of <5 or ≥5 CTCs CTCs/7.5 mL of blood in both time points—baseline and follow-up collection), using RUBYchip™ to isolate CTCs in MBC patients. Progression-free survival was calculated from the time of the baseline blood collection. Differences in survival curves were determined using the log-rank test.

Regardless of the isolation methodology, overall survival curves plateaued above 50% risk of survival, and sharp decreases to the probability of survival were observed only by the end of the study, indicating that a substantial proportion of patients survived for longer than the follow-up period, throughout the study. Although it may be indicative that there are very few patients at high risk at baseline, this small and heterogeneous cohort limits our ability to reach significant results based on one single time point. Nevertheless, accounting for CTC enumeration across the two time-points, the population was dichotomized as favourable when CTC counts were (i) below 5 CTCs at baseline and follow-up or (ii) above 5 CTCs at baseline, but below 5 CTCs at follow-up; and unfavourable when (i) above 5 CTCs at baseline and follow-up or (ii) below 5 CTCs at baseline, but above 5 CTCs at follow-up. The CellSearch^®^ detection method was not able to discriminate subpopulations in this cohort, since only a single patient was considered to have unfavourable CTC counts. Out of the 15 patients, using the RUBYchip™, discrimination of populations was well-defined with 40% that had favourable prognostic CTC counts and 60% with unfavourable CTC counts. Although no significant association between distinct subpopulations detected by RUBYchip™ and the survival measures was found (*p* = 0.3718, LogRank test), patients showing unfavourable CTC counts tended to have decreased survival (Figure 8).

### 2.8. Prognostic Value of HER2 Status in CTCs

HER2 status in CTCs was assessed across two time points; both baseline and follow-up collections were considered to dichotomize the population as concordant or discordant in relation to the molecular subtype assessed in tissue biopsy. The concordant subpopulation is in agreement with molecular subtyping performed on the primary tumour tissue, in both collections. The discordance in HER2 status was established considering a CTC HER2 status different from HER2 tissue assessment in at least one collection, with equal or over 50% in HER2 CTCs/Total CTCs ratio.

**Figure 9:**
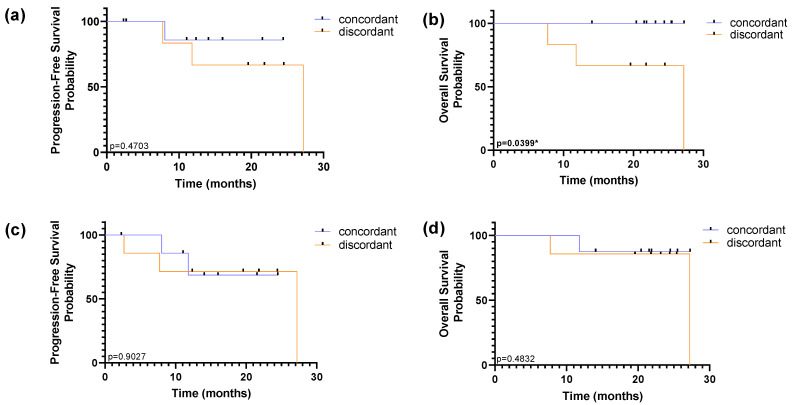
Kaplan–Meier curves of progression-free survival (PFS) and overall survival (OS) according to concordant or discordant HER2 status of CTC in relation to primary tissue assessment, using RUBYchip™ (**a**,**b**) and CellSearch^®^ (**c**,**d**) to isolate CTCs in MBC patients. PFS and OS were calculated from the time of the baseline blood collection. Differences in survival curves were determined using the log-rank test.

Using RUBYchip™ technology, concordance in HER2 status between CTCs and tissue biopsy was observed in 60% of the patients and discordance in 40%. Similarly, using CellSearch^®^, 53% of patients were found to be concordant and 47% discordant from tissue assessment. However, survival analysis showed no prognostic impact of the subpopulations detected by the CellSearch^®^, while the discordance of HER2 status in the tissue and the CTCs isolated with RUBYchip™ was associated with decreased PFS and OS (*p* = 0.4703 and *p* = 0.0399, respectively, using the log-rank test), as shown in Figure 9. Of note, this prognostic association was particularly significant for predicting OS, and it shows that patients considered discordant using the RUBYchip™ presented decreased OS, suggesting this technology enables discrimination of risk populations and its potential as a prognostic tool, even though the population size was limited.

## 3. Discussion

The RUBYchip™ has demonstrated a high capture efficiency (56% on average) at 120 μL/min, using breast cancer cell lines. This is due to its distinctive ability to directly filter CTCs from unprocessed whole blood samples, as well as the design features of the microfluidic chip, namely the pre-filters layout, prevented flow obstructions by eventual cell debris or microclots. The geometry of the RUBYchip™, along with its surface treatment, creates a favourable environment for CTC entrapment, eliminating most of the blood cells, such that a compromise between efficiency, speed and purity is obtained. The chip dimensions are ideal to allow deformable cells, generally blood cells, to pass through the filter gaps; however, cells that cannot deform, due to the smaller cytoplasm-to-nucleus ratio, are retained. These larger cells with bigger nucleus and cytoplasm are more likely to get trapped [86]. CTCs may have a wide range of cell sizes and phenotypes [87], as assessed using three cell lines different in cell size and phenotype aimed at testing a reliable representation of CTCs in circulation in clinical samples. This helps us better understand the capacity of the size-based filter to isolate CTCs, guaranteeing a compromise between cell isolation efficiency and purity at the best flow rate, despite the morphological differences that exist [86]. Despite cytomorphological differences between cell lines analysed, the results obtained were consistent for all the flow rates tested, and similar isolation efficiency values were observed for all the different cell lines. Moreover, the miniaturized chip size (fitting within a standard 25 × 75 mm glass slide) enables streamlined device fabrication and microscopy imaging.

Preclinical validation of the RUBYchip™ was achieved through a comparative study between our chip and the CellSearch^®^ system, the only FDA-approved technology for CTC enumeration. Side-by-side comparison of both technologies shows that RUBYchip™ consistently captured much more CTCs than CellSearch^®^, up to 10 times more, overall averaging 30 samples. The noticeable discrepancies in the number of CTCs isolated by the two different systems, in most of the samples (Figure 3), can be explained by the fact that the CellSearch^®^ system targets EpCAM+ CTCs only, missing out other CTCs with low EpCAM expression. Loss of CTCs during sample processing steps is also to be accountable, while whole-blood samples are introduced and filtered directly processed directly into the RUBYchip™, decreasing the possibility of cell loss.

In addition to the detection of epithelial-like CTCs (DAPI+/CK+/CD45−), DAPI+/CK−/CD45−/HER2+ cells were identified in the RUBYchip™, suggesting that this device is able to isolate cells with different phenotypes, increasing even further the number of isolated CTCs and providing additional important information of the disease status. In fact, other studies described the isolation of CK−/HER2+ CTCs in breast cancer recurring to liquid biopsy systems [53].

In previous clinical trials, using the CellSearch^®^ system, a threshold to distinguish patients with shorter progression-free survival and overall survival was established at ≥5 CTC/7.5. mL of blood [56]. In this study, 26 (86.7%) of the samples analysed by the CellSearch^®^ led to a classification of the patients as having good prognosis, since the number of CTCs was below the threshold. In contrast, the RUBYchip™ detected CTCs above the cut-off in 21 (70%) of the samples.

The fact that most of the patient samples analysed using CellSearch^®^ had a number of CTCs below threshold could lead to a misleading classification of the patients as having good prognosis; however, when comparing with the RUBYchip™ system, it was possible to observe that the same patient samples had a much higher number of CTCs, meaning that a misleading assumption concerning prognosis could have been made using CellSearch^®^. Even though the thresholds for each technology may differ, the results showed that the RUBYchip™ had a much better performance and allowed a more trustworthy evaluation of the CTC burden and, potentially, patient prognosis.

HER2 is an established therapeutic target in breast cancer. As such, screening its presence in CTCs provides information of great utility for therapeutic reasoning. Hence, the presence of HER2-positive CTCs in the patient samples was assessed by both technologies, and findings on CTC HER2 status were compared with the HER2 status of the tissue biopsy sample. Regarding the technologies in study, the results showed that not only was the RUBYchip™ able to isolate and detect HER2+ CTCs in a higher number of samples (9 patients in the RUBYchip™ against 4 patients in the CellSearch^®^), but also the number of HER2+ CTCs captured was higher, with an average of 2.6 CTCs, whereas the average number of HER2+ CTCs isolated by CellSearch^®^ was 1, proving once again that RUBYchip™ has a better performance.

The clinical reports on tissue biopsy analysis reported that four patients were diagnosed as having HER2+ tumours, and they were treated with anti-HER2 targeted therapy between the two time points. In these cases, tissue biopsy was made at the time of the primary diagnosis, which in some of these patients occurred several years before this study and may reflect an inaccurate assessment of the real-time HER2 status. The RUBYchip™ found HER2+ CTCs in all four patients with HER2 expression detected by tissue biopsy, while the CellSearch^®^ detected these cells in only one patient. It is important to mention that liquid biopsy analyses were performed blindly from the patients’ clinicopathological characteristics. Consensus among the three methodologies was found only in one patient (patient 1). The agreement between RUBYchip™ analysis and tissue biopsy classification supports the importance of CTCs isolation and characterization, to allow accurate diagnosis and subtype classification in real-time for successful therapy selection.

Since our results show that the number of HER2+ cells decreased between times of collection in patients under anti-HER2 targeted therapy, it may suggest a positive response to therapy. The higher number of CTCs in total by one of the patients (patient 1) may be indicative that the tumour burden is still high, but tumour phenotypical characteristics have changed, ideally even to a less invasive and more well managed subtype [88,89]. The presence of HER2+ cells, isolated by the RUBYchip™, in patients that were not diagnosed as having HER2+ tumours by the tissue biopsy analysis was observed. As mentioned in the introduction, cancer is a dynamic and heterogeneous disease [2]; hence, the molecular characteristics of the tumours can change over time due to therapeutic intervention or clonal evolution. As such, patients diagnosed with Luminal A (patients 2, 3, 7 and 9) or TNBC (patient 13) tumours could have suffered alterations over time, presenting HER2+ cells in the current collections as detected by the RUBYchip™. Additionally, it has been discussed that HER2 testing on the tissue biopsy in some circumstances may be unreliable or unrepresentative of the tumour [90]. It could be argued that these patients may have harboured HER2+ overexpression in the primary tumour; however, since the biopsy is performed to a small part of the tumour, it might not reflect the intratumour heterogeneity, and the presence of HER2 could have been missed. Tumour misclassification imposes risks to the patients, including decreased progression-free survival and overall survival [90,91].

Furthermore, to evaluate the role of CTCs in metastatic breast cancer, the correlation of CTC number and HER2 status with disease progression was studied. Five patients (33.3%) had increased CTC counts at follow-up collection. Patient 13, diagnosed with TNBC, increased his CTC counts three times. This increase can be an indicator of a possible resistance to therapy and worst prognosis. Patient 12, also diagnosed with TNBC and with metastases in the central nervous system, was only treated with radiotherapy and presented a considerable increase in CTCs. The increase in the number of CTCs between collections may be explained by the fact that brain metastases are the most aggressive and difficult to treat, correlated with a worst prognosis [92]. According to the clinical evaluation, regarding the disease progression and obtained from the CT/MRI scan, none of the patients had disease progression at the date of this submission (17 months after the first patient was recruited). Despite presenting a small cohort and a short follow-up, which did not allow a more longitudinal assessment of the patient’s evolution, the RUBYchip™ results showed that some patients had increased numbers of CTCs at the follow-up, which may be indicative of disease progression and worst prognosis. This hypothesis is in agreement with previous reports highlighting that liquid biopsy can provide information on disease progression even 1 year earlier than standard technologies used to monitor patients in the clinic [30]. Lastly, although the study involves a limited cohort of patients, CTC survival analysis results strengthen existing evidence reporting changes to HER2 status during the clinical course of metastatic BC. Most importantly, these results reinforce the need to use longitudinally collected samples to dynamically evaluate the heterogeneity of disease by HER2 status in CTCs, for which tissue sampling still bears several limitations, as opposed to the real-time CTC repetitive sampling. Additionally, the sensitivity of the CTCs detection method is important to enable discrimination of patient populations that present a poor evolution of the disease, since low-sensitivity technologies miss the opportunity to distinguish prognostic groups and therefore fail to contribute to the clinical management of patients, including treatment redirection.

## 4. Materials and Methods

### 4.1. Microfluidic Device Design and Fabrication

The RUBYchip™ is the fourth generation (following the CROSS chip [32]) of a microfluidic system that comprises an inlet and an outlet flow channel and is designed to split the blood equally in 2 different areas, each area displaying 4 separated modules that are 17.5 mm wide; these have the capacity to process a total of 7.5 mL of blood.

**Figure 10:**
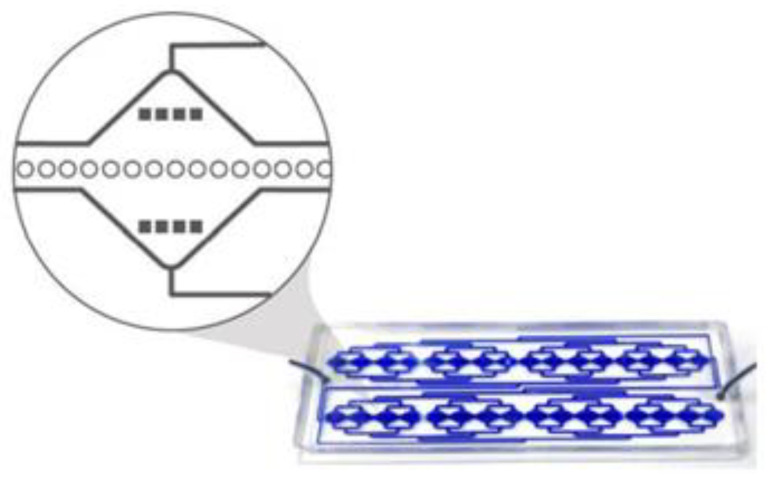
The RUBYchip™ is designed to capture CTCs based on physical properties such as size and cellular deformability. Filtering areas are composed by interspaced micropillars, distanced from each other in 5 µm.

Each module has in its middle section a plurality of anisotropic micropillars layout in a single row, with 25 μm diameter and interspaced in about 5 μm, thereby forming a plurality of gaps which form the cell filtering area. The size, geometry and aspect ratio of the micropillars, as well as the gap size, were carefully chosen to allow blood cells to deform and gently flow through, retaining, however, larger and more rigid cells in the filter. Additional pre-filters are part of the design, and these have 120 μm gaps to prevent clumps or debris from blocking the device. Each microfluidic device holds an approximate internal volume of 50 μL (Figure 10).

The microfluidic masters were designed in 2D AutoCAD software (Autodesk AutoCAD 2020, Autodesk inc., CA, USA) and fabricated in a 200 mm silicon wafer using photolithography and deep reactive ion etching. Briefly, the silicon wafer (P/Boron, <100>, Siegert Wafer GmbH, Aachen, Germany) was patterned using a Direct Write Laser system (DWL 2000 Heidelberg Instruments, Heidelberg, Germany). The pattern was then etched with sulphur hexafluoride (SF6, Sigma Aldrich/ Merck KGaA, Darmstadt, Germany) by Silicon Deep Reactive Ion Etching (SPTS Pegasus, SPTS Technologies Ltd., Newport, England). Trench depth was measured in between steps using an optical profilometer (Hyperion, OPM profilometer, OPM Messtechnik GmbH, Ettlingen, Germany) until 20 μm depth was reached. Residues were stripped using oxygen plasma, and the master was characterized by Scanning Electron Microscopy (NovaNanoSEM, FEI, Oregon, USA). Finally, the wafer was diced (Automatic Dicing Saw, DAD3350, DISCO HI-TEC FRANCE SARL, Gardanne, France) into the individual masters, cleaned with isopropyl alcohol (IPA, Sigma Aldrich) and deionized water and dried at 150 °C on a hot plate.

Before replication, the masters were hydrophobized with a vapor-phase treatment in trichloro(1H,1H,2H,2H-perfluorooctyl)silane (Sigma Aldrich) for two hours (one hour at 65 °C).

After hydrophobization, for rapid prototyping using soft lithography, polydimethylsiloxane prepolymer was mixed with a cross-linker (PDMS, SYLGARD^TM^ 184 Silicone Elastomer, Dow Chemical Company, Ellsworth Adhesives, Madrid, Spain) at 10:1 ratio, degassed, and poured over the master and cured at 65 °C for 2 h. Subsequently, the PDMS replica was unmoulded, and both inlet and outlets were punched. Finally, microscope glass slides (size 25 × 75 mm, ThermoFisher Scientific, Darmstard, Germany) and the PDMS replicas were treated with oxygen plasma (Plasma Cleaner PDC-002-CE, HarrickPlasma, NY, USA) to produce irreversible bonding.

To conclude the device preparation, the chips were connected to a syringe pump (NE-1200, New Era Syringe Pumps, Tecan France, Lyon, France) and filled with 350 μL of ethanol (Sigma Aldrich) at 100 μL/min, 350 μL of 10 mM phosphate buffer saline (PBS, Sigma Aldrich) at 120 μL/min, and 4000 μL of 1% Pluronic F-127 (Sigma Aldrich) at 140 μL/min for coating and preventing cell attachment onto the channel surface.

### 4.2. Optimization Studies

#### 4.2.1. Cell Culture and Spiking Experiments

The human cancer cell lines MCF-7 (ATCC, CRL-3435), MDA-MB-435 (ATCC, HTB-129) and SKBR3 (ATCC, HTB-30) were used for this study. Both MDA-MB-435 and SKBR3 cell lines were cultured in Dulbecco’s Modified Eagle’s Medium (DMEM, Gibco, ThermoFisher Scientific, Darmstard, Germany), supplemented with 10% foetal bovine serum (FBS, Gibco) and 1% penicillin/streptomycin (Pen/Strep, Corning, NY, USA). For MCF-7 cell culture, additional 0.1% human insulin (Sigma Aldrich) supplementation was provided. All the cell lines were cultured as a monolayer at 37 °C in a 5% CO_2_ humidified atmosphere.

To assess the isolation efficiency of the RUBYchip™, the three different cell lines were used in spiking experiments, as follows. Two hundred cells stained with Hoechst for 30 min were spiked in 7.5 mL of whole blood collected from healthy donors. The cells were injected in the RUBYchip™ using a syringe pump at four different flow rates: 100, 120, 140 and 160 μL/min. Trapped cells were rinsed with 350 μL of 2% bovine serum albumin (BSA, Sigma Aldrich) in PBS, fixed with 350 μL of 4% paraformaldehyde (PFA, Sigma Aldrich) for 20 min at room temperature (RT) and washed with PBS. The analysis of the trapped cells was performed using a fluorescence inverted Nikon TI-E microscope. To determine the isolation efficiency of the RUBYchip™, the number of Hoechst-positive cells trapped in the device was compared with the total number of cells spiked, as in Equation (1). Experiments were done in triplicate.

(1) $$CTC\;isolation\;Efficiency\;\left(\%\right)=\frac{Trapped\;CTCs}{Spiked\;cells}\times 100$$

#### 4.2.2. Immunocytochemistry Experiments

Immunocytochemistry (ICC) studies were performed in order to characterize the cells trapped in the RUBYchip™. Different experimental conditions were tested to optimize antibody staining conditions. These studies were done both in well plate and in the microfluidic device. Negative control studies were performed in device using healthy volunteers’ samples. Conjugated antibodies were used, meaning that direct IF studies were performed, in order to optimize the time of preparation and incubation of the antibodies, since protocols for direct IF are usually shorter as they only require one labelling step, and to minimize the risk of cross contamination.

The panel of antibodies selected included monoclonal Anti-Cytokeratin pan-FITC antibody (clone C-11, recognizes human cytokeratins 4, 5, 6, 8, 10, 13 and 18; Sigma Aldrich; F3418; 1.9 mg/mL) to target epithelial cells; DyLight 550 Anti-ErbB2/HER2 (Immunostep^®^, Salamanca, Spain; 1399990570; 0.85 mg/mL) to target cells with HER2 overexpression; Cyanine5 Anti-Human CD45 (Immunostep^®^; 1399990730; 1 μg/mL) to label blood cells (used as a CTC exclusion criteria); and DAPI (Sigma Aldrich; D9564; 1 mg/mL) to stain the nucleus. Using a fluorescence inverted Nikon- TI-E microscope, and using NIS^®^ Software, it was possible to obtain multi-channel fluorescence images, with DAPI, CK, HER2 and CD45, in the blue, green, orange and red channels, respectively.

For the ICC performed in well plate, MCF-7, MDA-MB-435, SKBR3 and PBMCs were used. Cell lines were seeded on sterile glass coverslips (treated with Poly-Lysine) and allowed to grow for 24 h. PBMCs were isolated from whole blood from healthy donors by density gradient centrifugation using Histopaque^®^ (Sigma Aldrich). For immunofluorescence staining, the cells were incubated with the antibodies described above, at the dilutions to be tested (Table 4).

The same cell lines were used in the ICC performed in device; however, instead of isolated PBMCs, whole blood from healthy donors was spiked with cell lines to mimic the processing conditions of cancer patient samples. A total of 7.5 mL of whole blood samples were spiked with 200 cells of each cell line, using a working cell suspension of 100 cells/100 μL, and pumped at 120 μL/min in the RUBYchip™ using a syringe pump. Whole blood from three different healthy donors pumped at 120 μL/min in the RUBYchip™ was used as negative control in ICC experiments.

After cell isolation and staining, images of the device were acquired using a Nikon-Ti-E microscope, in 8 different large scans of 25 fields-of-view each, dividing the 2 rows of the device in 4 different areas. Optimization assays were made until an ideal antibody dilution was achieved.

In order to have an expression negative control, where no cancer cells are present, whole blood from healthy donors was stained alone with the same selection of antibodies, and 7.5 mL of blood was collected from three different healthy donors. The blood was pumped at 120 μL/min in the RUBYchip™ using a syringe pump. Once sample processing was finished, standard ICC protocol was performed as described above.

### 4.3. Patient Sample Collection and Processing

In the present study, 15 patients diagnosed with metastatic breast cancer were recruited at the Hospital de Santa Maria between November 2018 and September 2019 and followed according to the Oncology Department guidelines for disease evaluation. This study was ethically approved by the local institutional review board at iMM and complied with all national regulations. All patients participated voluntarily after providing written informed consent. The study was conducted in accordance with the Declaration of Helsinki and good clinical practice guidelines.

**Figure 11:**
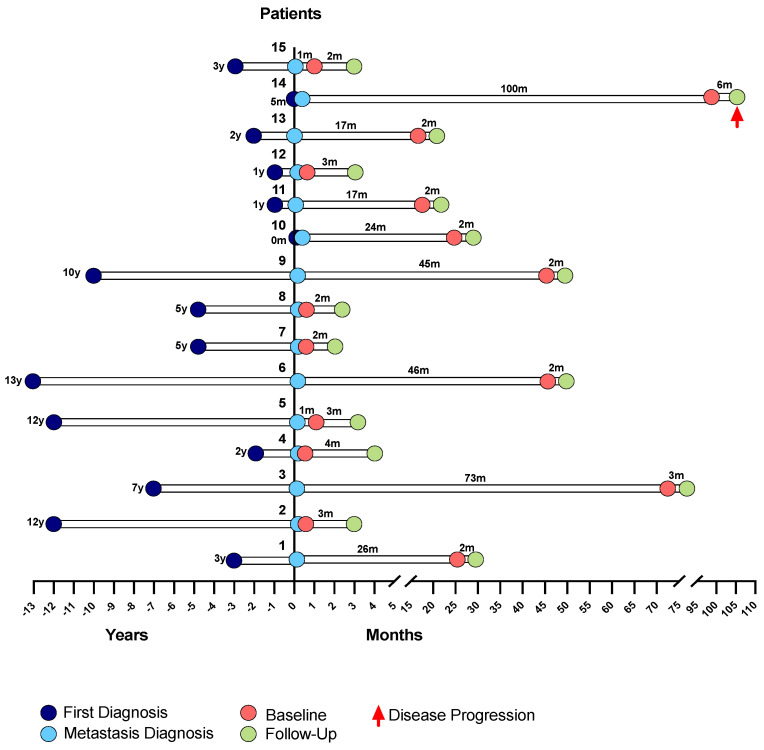
Representative chronogram of the different moments under study, namely: metastasis diagnosis, baseline collection and subsequent follow-up collection, for each patient.

Clinicopathological information was recorded for all patients. As previously mentioned, each patient had blood collections at two time points, at a baseline and at monitoring follow-up, after 12 weeks of ongoing treatment (Figure 11).

Fifteen millilitres of blood was collected from each patient in order to process the samples with both technologies in parallel. CellSave^®^ preservative tubes were used to collect blood samples for CellSearch^®^ analysis (Menarini-Silicon Biosystems, Bologna, Italy), while EDTA-coated tubes were used for sample collection for the RUBYchip™. All the samples were anonymized and encoded prior to analysis.

Processing by the RUBYchip™ was done within 4 h of collection, at the Molecular Medicine Institute (IMM), and then fixed and shipped to the International Iberian Nanotechnology Laboratory (INL) within 24 h to be analysed. The samples to be processed by CellSearch^®^ were shipped in preservative tubes at RT to the Liquid Biopsy Analysis Unit of the Health Research Institute of Santiago (IDIS, Spain) and processed within 96 h, following the protocol of the manufacturer.

### 4.4. CTC Isolation and Characterisation Using the RUBYchip™

Cells from patient samples isolated using the RUBYchip™ were fixed with 4% PFA for 20 min, permeabilized with 0.25% Triton X-100 (Sigma Aldrich) and blocked with 2% BSA. Isolated cells were fluorescently labelled inside the device with the antibody panel previously mentioned, and images were obtained using a fluorescence inverted Nikon- TI-E microscope. Recurring to the CK, CD45, HER2 and DAPI immunostaining as well as to morphological properties, such as the nucleus size, the criteria to classify cells were developed. After excluding debris, irregular fluorescence shapes or with a dark outline, the clear events with cell-like morphology were classified.

To achieve CTC counts, cells isolated in the RUBYchip™ were manually enumerated, and randomized blind analysis was performed. Cells classified as DAPI+/CK+/CD45− or DAPI+/CK+/HER2+/CD45− were considered for CTCs enumeration. The presence of cells showing HER2 overexpression was also tested by confirming the presence of DAPI+/CK+/HER2+/CD45− cells (Table 5). The number of CTCs isolated by the RUBYchip™ was verified and validated by a technical expert routinely involved in the analysis of CellSearch^®^ data.

### 4.5. CTC Isolation and Characterisation Using CellSearch^®^ System

A total of 7.5 mL of blood was employed for CTCs enumeration by the CellSearch System, using CellSearch Epithelial Circulating Tumour Cell Kit (Menarini, Silicon Biosystems Inc, Huntington Valley, PA, USA). This system automatically immunoisolated EpCAM+ CTCs, incubating the blood with ferrofluids coated with an anti-EpCAM antibody (clone VU1D9). Before this incubation, 7.5 mL of blood was centrifuged at 600× *g* for 10 min at RT. The system removed the plasma fraction and incubated the cell fraction with the ferrofluids. After the isolation using a magnetic field, the system labelled the enriched cells with phycoerythrin (PE) conjugated anti-cytokeratins (CKs, 8, 18 and 19) antibodies, with allophycocyanin (APC) conjugated anti-CD45 antibodies, FITC-labelled anti-HER2 (CellSearch tumour phenotyping reagent HER2; (Menarini, Silicon Biosystems Inc, Huntington Valley, PA, USA) and with 4,6-diamino-2- phenylindole (DAPI) to identify the nucleus. The CellTracks Analyzer (Menarini, Silicon Biosystems Inc, Huntington Valley, PA, USA) was then used to acquire digital images of the four different fluorescent dyes using a 12-bit camera. These images were reviewed by trained operators in order to determine the CTC count. Only round/oval, intact DAPI+, CK+ and CD45− cells were considered as CTCs. HER2 status on the CTCs was determined according to specific criteria described by [93], scoring the HER2 intensity as negative (0), low (1), moderate (2) and high (3).

### 4.6. Statistical Method

Statistical analysis was performed using GraphPad Prism software, version 8 (GraphPad Software, San Diego, CA, USA).

The Wilcoxon test (non-parametric inference statistical test) considering a 95% confidential interval was used to compare CTC enumeration using CellSearch^®^ versus the RUBYchip™ technologies in the same metastatic breast cancer patient. The results were considered statistically significant if the *p*-value was less than 0.05.

Kaplan–Meier curves were based on three different dichotomizations: (1) based on the number of circulating tumour cells (cut-off as ≥5 CTC) at baseline; (2) favourable or unfavourable patients, considering CTC count (≥5 CTC) in both time points (baseline and follow-up collections); and (3) based on concordance of HER2 status between CTCs and primary tumour tissue. Progression-free survival (PFS) and overall survival (OS) were determined as the time between the baseline date and the date of next clinical progression or death. Data were censored at last follow-up if progression or death had not occurred. Differences in survival curves were determined using the log-rank test.

## 5. Conclusions

This study demonstrates the reliable, efficient and sensitive detection of CTCs in metastatic breast cancer by using a novel microfluidic device, the RUBYchip™. The performance of the RUBYchip™ was tested in spiked cancer cells in healthy volunteer blood samples as well as in clinical patient samples. Concerning CTC capture and enumeration in patient samples, the RUBYchip™ demonstrated superior performance when compared to the gold standard technology used in the clinic, CellSearch^®^, in both moments of collection. The main reason behind this outcome is the choice of the isolation strategy regardless of the cell phenotype, based on the size and deformability of cells, used by the RUBYchip™. In contrast, CellSearch^®^, which bases cell selection on the expression of epithelial proteins in the membrane of cancer cells, compromises the isolation of cells not expressing those specific receptors. Additionally, the RUBYchip^TM^ isolates CTCs in the sample with no need of any sample pre-treatment, known to reduce the already scarce number of CTCs in the sample, which in turn contributes to having a higher efficiency as well as increased potential of yielding feasible numbers of viable CTCs.

The demonstrated higher capture efficiency of the RUBYchip^TM^ proved to be fundamental in the accurate assessment of HER2 status in CTCs isolated from metastatic breast cancer samples. Regarding HER2 testing, the RUBYchip^TM^ allows to overcome limitations of tissue biopsy histopathological assessment, if used concomitantly to the latter to dynamically assess the presence of HER2+ CTCs. Furthermore, if used at subsequent monitoring time-points, it allows for the real-time HER2 analysis of histologically HER2-positive MBC patients under targeted therapy. In selected patients, the RUBYchip^TM^ detected a shift in HER2 status from positive at baseline to negative at follow-up, in response to anti-HER2 treatment.

Despite the fact the study presents a small cohort of 15 patients, these preliminary findings concerning the use of RUBYchip^TM^ in a clinical setting are indicative of tangible added potential for clinical application in MBC. This study paves the way towards larger multi-centre clinical trials, an imperative next step in assessing the putative clinical utility of employing such technologies in the management of the metastatic disease and contribute to assist in clinical decision-making.

## 6. Patents

The RUBYchip™ design is based on the patent PCT/EP2016/078406, filed with some of the authors in front of the EPO on 22 November 2016, covering the geometry of the microfluidic system for CTC isolation.

## Figures and Tables

**Table 1:** Clinicopathological characteristics of patients enrolled in this study.

Patient Clinical Data		
	N	%
Cohort		
Number of patients	15	--
Number of collections	30	--
Sex		
Female	15	100
Age at MBC diagnosis (years)		
Average (range)	47 (29–65)	
≥60 years	3	20.0
<60 years	12	80.0
HER2 status ^a^		
Positive	4	26.7
Negative	11	73.3
BC subtype ^b^		
TNBC	1	6.7
ER+/PR−/HER2−	3	20.0
ER+/PR+/HER2−	7	46.7
ER+/PR−/HER2+	2	13.3
ER+/PR+/HER2+	2	13.3
Grade		
II	10	66.7
III	4	26.7
Unknown	1	6.7
Metastatic site, number		
single	12	80.0
multiple	3	20.0
Metastatic site		
Visceral ^c^	6	40.0
Bone	5	33.3
Multiple/other	4	26.7
Treatment initiated at baseline ^d^		
Including Chemotherapy	5	33.3
Including Hormone therapy	8	53.3
Including HER2-targeted therapy	3	20.0
Including other targeted therapy	5	33.3
Radiotherapy only	1	6.7
MBC diagnosis at inclusion		
Relapse	14	93.3
De novo	1	6.7
Vital Status, at conclusion		
Alive with metastatic disease	15	100
CTC positive, at baseline		
RubychipTM	15	100
CellSearch^®^	7	46.7
CTC positive, at follow up ^e^		
RubychipTM	14	93.3
CellSearch^®^	4	26.7

^a^. HER2 status at the time of study inclusion; ^b^. No information was available from the metastasis, and the subtype was derived by staining of the primary tumour; ^c^. Visceral metastasis defined as lung, liver, brain, lymph nodes, peritoneal and/or pleural involvement; ^d^. Treatment initiated = first or new line of treatment initiated at baseline collection; ^e^. clinical follow up at 3 months after baseline.

**Table 2:** Results of the CTC count using RUBYchip™ versus CellSearch^®^.

		Baseline (*n* = 15)		Follow-Up (*n* = 15)		Overall (*n* = 30)	
		RUBYchip™	CellSearch^®^	RUBYchip™	CellSearch^®^	RUBYchip™	CellSearch^®^
CTCs/7.5 mL	Average	12	2	8	0	10	1
	Median	6	0	6	0	6	0
N° of cases	CTC +	15 (100%)	7 (47%)	14 (93%)	4 (27%)	29 (97%)	11 (37%)
	CTC ≥ 5	12 (80%)	3 (20%)	9 (60%)	1 (7%)	21 (70%)	4 (13%)
	CTC < 5	3 (20%)	12 (80%)	6 (40%)	14 (93%)	9 (30%)	26 (87%)

**Table 3:** Number of CTCs isolated by the RUBYchip™ and the CellSearch^®^ in the 15 metastatic breast cancer patients, at both time points.

Patients	CTC Enumeration			
	RUBYchip^TM^		CellSearch^®^	
	Baseline	Follow-Up	Baseline	Follow-Up
1	15	23	2	1
2	4	2	0	0
3	26	1	1	0
4	24	0	0	0
5	12	1	7	0
6	5	7	0	1
7	6	3	0	0
8	6	1	0	0
9	28	10	0	0
10	6	6	28	56
11	3	15	1	0
12	3	12	0	0
13	5	20	1	1
14	25	14	13	0
15	6	6	0	0

**Table 4:** Working dilutions tested for each of the selected antibodies in the ICCs performed.

ICC	DAPI	CK	HER2	CD45
performed in well plate	1:5000	1:200	6 μg/mL (1:142)	1:50
performed in Device (I)	1:5000	1:300	6 μg/mL (1:142)	1:50
performed in Device (II)	1:5000	1:400	6 μg/mL (1:142)	1:100
performed in Device (III)	1:5000	1:600	6 μg/mL (1:142)	1:200
Negative Controls	1:5000	1:200	6 μg/mL (1:142)	1:50
Patient Samples	1:5000	1:200	8 μg/mL (1:106)	1:100

**Table 5:** Immunofluorescent staining characteristics for identifying CTCs.

Characteristic	Cell Classification
Irregular shape or dark outline	Debris
DAPI+/CK+/HER2−/CD45−	CTC
DAPI+/CK+/HER2+/CD45−	
DAPI+/CK−/HER2−/CD45+	WBC
DAPI+/CK−/HER2+/CD45+	
DAPI+/CK+/HER2−/CD45+	Favour WBC
DAPI+/CK+/HER2+/CD45+	
DAPI+/CK−/HER2+/CD45+	

## Data Availability

Data sharing is not applicable to this article.
